# CLET: Computation of Latencies in Event-related potential Triggers using photodiode on virtual reality apparatuses

**DOI:** 10.3389/fnhum.2023.1223774

**Published:** 2023-09-19

**Authors:** Piyush Swami, Klaus Gramann, Elise Klæbo Vonstad, Beatrix Vereijken, Alexander Holt, Tomas Holt, Grethe Sandstrak, Jan Harald Nilsen, Xiaomeng Su

**Affiliations:** ^1^Motion Capture and Visualization Laboratory, Applied Information Technology Group, Department of Computer Science, Norwegian University of Science and Technology, Trondheim, Norway; ^2^Section for Visual Computing, Department of Applied Mathematics and Computer Science, Technical University of Denmark, Kongens Lyngby, Denmark; ^3^Biomedical Engineering Techies, Broendby, Denmark; ^4^Biological Psychology and Neuroergonomics, Technical University of Berlin, Berlin, Germany; ^5^Department of Neuromedicine and Movement Science, Norwegian University of Science and Technology, Trondheim, Norway

**Keywords:** motion-capture (Mocap), latencies, electroencephalography (EEG), event-related potential (ERP), interface

## Abstract

To investigate event-related activity in human brain dynamics as measured with EEG, triggers must be incorporated to indicate the onset of events in the experimental protocol. Such triggers allow for the extraction of ERP, i.e., systematic electrophysiological responses to internal or external stimuli that must be extracted from the ongoing oscillatory activity by averaging several trials containing similar events. Due to the technical setup with separate hardware sending and recording triggers, the recorded data commonly involves latency differences between the transmitted and received triggers. The computation of these latencies is critical for shifting the epochs with respect to the triggers sent. Otherwise, timing differences can lead to a misinterpretation of the resulting ERPs. This study presents a methodical approach for the CLET using a photodiode on a non-immersive VR (i.e., LED screen) and an immersive VR (i.e., HMD). Two sets of algorithms are proposed to analyze the photodiode data. The experiment designed for this study involved the synchronization of EEG, EMG, PPG, photodiode sensors, and ten 3D MoCap cameras with a VR presentation platform (Unity). The average latency computed for LED screen data for a set of white and black stimuli was 121.98 ± 8.71 ms and 121.66 ± 8.80 ms, respectively. In contrast, the average latency computed for HMD data for the white and black stimuli sets was 82.80 ± 7.63 ms and 69.82 ± 5.52 ms. The codes for CLET and analysis, along with datasets, tables, and a tutorial video for using the codes, have been made publicly available.

## 1. Introduction

### 1.1. Motivation

Many applications of electroencephalography (EEG) and event-related potentials (ERP) (Luck, 2012; Nidal and Malik, 2014) require the use of triggers (or tagging) (Wang et al., 2016; Cattan et al., 2018) to indicate the exact onset of presented stimuli (mostly visual or auditory events) (Miyakoshi et al., 2021; Ignatious et al., 2023) so that the recorded physiological data can be synchronized. However, variability in various hardware and software typically lead to differences in latencies between the transmission and reception of triggers (Wang et al., 2016; Cattan et al., 2021; Iwama et al., 2022). It is of importance that these latencies in triggers should not be confused with another type of latency that is present in the neural markers, such as N170, P250, N400, etc. (Luck, 2012; Cattan et al., 2021; Miyakoshi et al., 2021; Abreu et al., 2023). Although there are several studies (Hoormann et al., 1998; Kiesel et al., 2008; Wu et al., 2013; Liesefeld, 2018; Ignatious et al., 2023) that showcase the computation of latencies in neural markers and their association with brain activities, the focus of the current work is on the computation of latencies in event-related potential TRIGGERS, which is a critical pre-processing step for any brain-computer interface (BCI) study. Existing literature (discussed in the next section) lacks a methodical approach with datasets to compute trigger latencies, especially for immersive virtual reality (VR) apparatus. The objective to overcome this knowledge gap formed the main motivation of this work.

### 1.2. Literature survey

While past studies (Cattan et al., 2018; Iwama et al., 2022) have highlighted shifting of ERP epochs based on computed average latencies, other studies illustrate details about setting up the triggers to overcome latency differences (Wang et al., 2016; Cattan et al., 2018). To the best of our knowledge, only a few studies exist which describe the importance and considerations for computing the latencies in triggers using immersive VR systems (Wang et al., 2016; Cattan et al., 2021; Iwama et al., 2022). In the literature (Wang et al., 2016; Lees et al., 2018), Rapid Serial Visual Paradigm (RSVP) (Wang et al., 2016; Lees et al., 2018) is one of the common, simple, yet effective approaches for sending triggers. Lab Streaming Layer (LSL) (Stenner et al., 2023) is another preferred choice in many studies (Wang et al., 2016; Iwama et al., 2022). The availability of open-source resources like Simulation and Neuroscience Application Platform (SNAP) (Kothe, 2023) developed in Python to ease stimuli presentation, also favored using LSL. However, this could mandate using a setup with bigger memory and displays with higher refresh rates compared to the RSVP approach (Wang et al., 2016). For efficient hardware-software synchronization with the LSL approach, an extra hardware setup like Light Diode Resistor Comparator Circuit (LDRCC) (Wang et al., 2016) has also been used. Hence, the RSVP approach with C# programming was followed in this work.

Most of the existing literature (Lopez-Calderon and Luck, 2014; Cattan et al., 2018, 2021; Lees et al., 2018; Williams et al., 2021; Huang et al., 2022; Iwama et al., 2022), which at least provide scattered details and some considerations for settings up triggers, are based on using only EEG sensors or at most a few auxiliary (AUX) sensors. Although synchronization with other modalities like motion-capture (MoCap) has been achieved (Miyakoshi et al., 2021), the knowledge about setting up its triggers and computation of latencies during VR experiments is lacking.

### 1.3. Objectives

The work present work contributes to overcoming the existing knowledge gaps in the literature by setting up the following objectives: (1) to demonstrate the Computation of Latencies in Event-related potential Triggers (CLET) as a tool for measuring latencies in multi-model experiments, especially when VR apparatuses are used; and (2) to provide open access to novel datasets, codes, tables, and a tutorial video^1^ to ensure transparency and reproducibility of results, as well as boost future improvements in the algorithms.

### 1.4. Brief outline of the next sections

The work was performed to test the synchronization of triggers in a multi-model experiment that was designed to monitor biomechanics (specifically gait patterns) and physiological signals. This article is limited to the illustration of the CLET approach. The experimentation is explained in the next section, and the proposed method is detailed in the subsequent section. The computed latencies and their distribution are described in the results section, with the method’s advantages compared to the state-of-art being covered in the discussion section. Finally, the conclusions, limitations and future scope for improvement are described in the last section.

## 2. Methods

### 2.1. Experimentation

**FIGURE 1:**
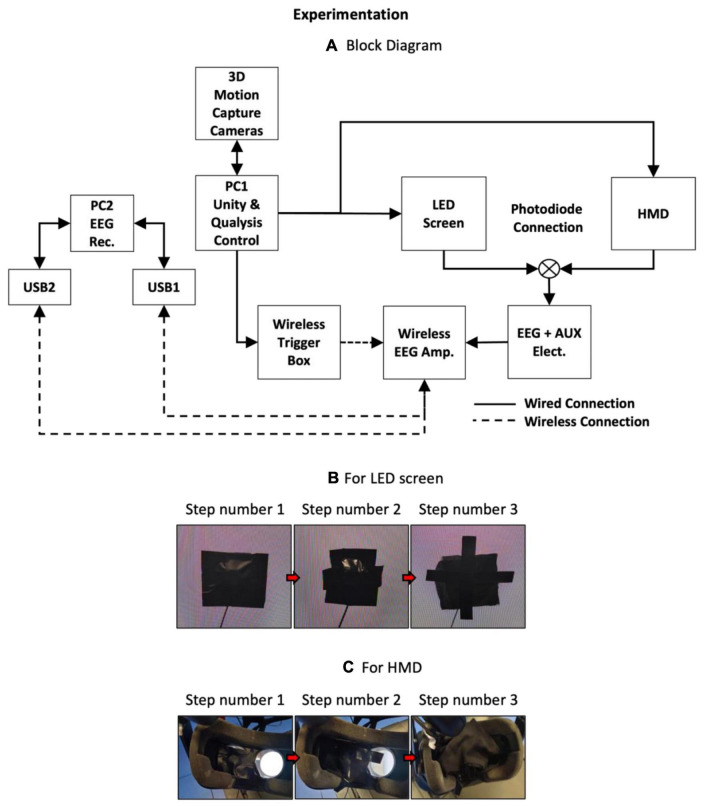
**(A)** Block diagram showing the experimental setup. Procedure to place and cover photodiode on **(B)** Light Emitting Diode (LED) screen, and **(C)** left eyepiece of a head-mounted display (HMD). For both displays, step number 1 is to cover the photodiode with the black tape. Step number 2 is to cover the tape with a piece of black cloth and secure the cloth. Step number 3 is to repeat the last step. Although the experiment was conducted in a dark room, the above steps ensured that any ambient light, if present due to displays or other electronics, did not affect the photodiode signals.

The experiment setup with the rapid serial visual paradigm (RSVP) is shown in Figure 1A. The apparatus included one desktop personal computer (PC1)–with Unity and Qualisys Track Manager (QTM) software installed and one laptop (PC2) with EEG Recorder (Brain Vision Recorder) software installed. The biomechanics monitoring setup included 3D MoCap cameras (nine Qualisys high-speed cameras and one Miqus camera), and the physiological monitoring setup included Brain Products LiveAmp with 64-channel wireless EEG with dual channels EMG and one PPG sensor. A photodiode was connected as an auxiliary (AUX) sensor with either a LED screen or an HMD at a time. The LED screen consisted of a Sony TV (KDL-75W855C) with dimensions of 167.7 × 96.9 × 7.9 cm and a refresh rate of 100 Hz. The HMD consisted of HTC VIVE Pro Eye with a field of view of 110° and a refresh rate of 90 Hz.

As shown in Figure 1A, PC1 was used to control the MoCap apparatus through a wired connection with the QTM software. The same computer was also used for stimulus presentation through the Unity software, which sent triggers to the EEG amplifier (amp.) unit via a USB connection to the wireless trigger box. Both displays were also connected to PC1. Data recorded using the amplifier was sent via Bluetooth to USB1 and USB2 dongles connected to PC2. The stimuli consisted of ∼100 images of black and white colors. The neutral image consisted of a gray color image with a red-colored cross at the center. The selection of these images represented ON (white screen) and OFF (black screen) input signals to the photodiode placed on the display. This procedure aligned with the protocol described in Cattan et al. (2021). The inter-stimulus interval (*isi*) was randomly varied between 1.0–1.5 s with a fixed stimulus duration of 0.3 s. The experimental paradigm was written in C# inside the Unity software. Here, the white and black stimuli were assigned to be displayed as “S1” and “S2” triggers, respectively, in EEG recordings. Similarly, the start and end of the recordings were assigned “S7” and “S8” triggers, respectively.

**FIGURE 2:**
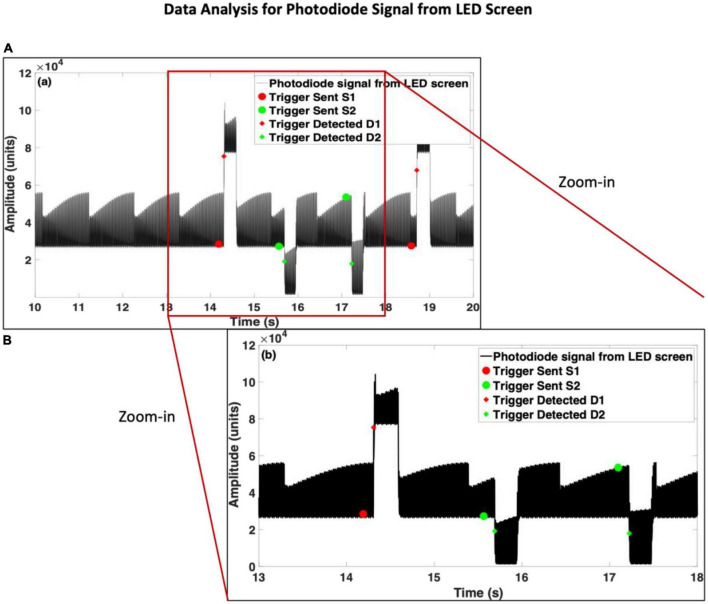
Photodiode data recorded from Light Emitting Diode (LED) screen. Panel **(A)** is scaled to an instance of 10 s data, and panel **(B)** is scaled to an instance of 5 s data, to show changes in the shape of the signal after the onset of each type of stimulus.

**FIGURE 3:**
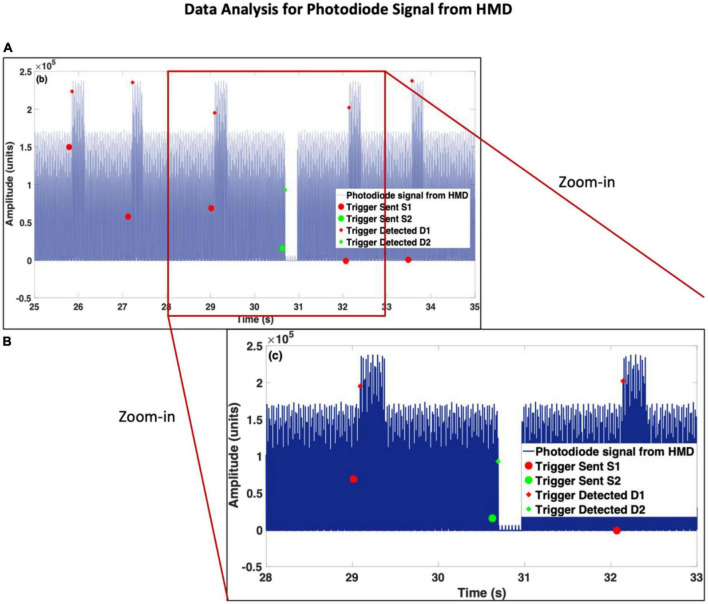
Photodiode data recorded from the head-mounted display (HMD). Panel **(A)** is scaled to an instance of 10 s data, and panel **(B)** is scaled to an instance of 5 s data, to show changes in the shape of the signal after the onset of each type of stimulus.

The photodiode was placed on the LED screen and covered with a black tape and then black cloth to prevent any disturbance from any external light source. The procedure to place and cover the photodiode for both displays is shown in Figure 1B. The entire experiment was performed in a dark room. After the calibration of Qualisys markers, the test involved recording QTM, then the Brain Vision Recorder (BV Rec.), followed by Unity. When the test was complete, a text notification was visible in the Unity console window. The operator was required to stop the software in the reverse order, i.e., Unity, then BV Rec., followed by QTM. The data was synchronized through triggers and time points noted in the log files. A similar process was repeated by the placement of the photodiode on the HMD and covering the sensor with black tape and black cloth. The procedure for placing the photodiode sensor is shown in Figure 1C. Each test lasted ∼5 min. The photodiode data recorded from each display is shown in Figures 2, 3. For better visualization of its shape, a section of 5 s is shown in Figures 2B, 3B. The methods developed to analyze the photodiode data recorded (see next section) have subtle variations for each of the displays due to the differences in their shapes.

### 2.2. Data analysis

#### 2.2.1. Prerequisites

The algorithm was developed in MATLABBR2021a using inbuilt functions except for pop_fileio() (available in open-source EEGLAB library), which was used to load data.

#### 2.2.2. Algorithm for CLET using data recorded from the LED screen

*Notations:* Let triggers sent for the first type (white image) and the second type (black image) of stimuli be *S*1 and *S*2, respectively. Let triggers detected for the first type (white image) and the second type (black image) of stimuli be *D*1 and *D*2, respectively.

*Inputs:* Directory Path, File Name, the Lower limit of the inter-stimulus interval (*Lisi*, i.e., 1 s in this study), Thresholds *ThD*1 and *ThD*2 for detecting triggers for the first and the second type of stimuli, respectively. For the LED screen, *ThD*1 is the transient rise in the signal’s amplitude (Figure 2B) above which the algorithm would detect *D*1. Similarly, *ThD*2 is the transient drop in the signal’s amplitude (Figure 2B) below which the algorithm would detect *D*2. The developed code would first plot the photodiode data extracted from the .eeg file. Then the user would be required to visually inspect and define any one of the 100 values of *D*1 and *D*2. These values do not need to be precise. For the data shown in Figure 2, the value of *ThD*1 = 60000 and *ThD*2 = 20000.

*Outputs:* Array of onset time (s) when triggers were sent *tS*1 and *tS*2; array of onset time (s) when triggers were detected *tD*1 and *tD*2; array of latencies between *D*1 and *S*1, i.e., *LatD*1*S*1 (ms), and array of latencies between *D*2 and *S*2, i.e., *LatD*2*S*2 (ms).

*I. Steps:*

1. Load photodiode data.
2. The gap between the indices of the detected triggers indxGap = Sampling rate fs ∗ Lisi
3. To compute *LatD*1*S*1:
3.1. Define matrix *PosPhoto* containing 1’s and 0’s where 1’s represent positions of positive peaks *PosPhoto* = *DataPhoto* ≥ *ThD*1
3.2. For *PosPhoto* = *P*_1_,*P*_2_,…,*P*_*n*_; find the difference between the (*n* + 1) − *n* terms, i.e., *PosDiff* = (*P*_2_ − *P*_1_), (*P*_3_ − *P*_2_), …, (*P*_*n* + 1_ − *P*_*n*_)
3.3. Pad 0 in the beginning, ∴*PosDiff* = 0, *PosDiff*
3.4. Indices for *D*1 *indxD*1 = *find* (*PosDiff* = = 1)
3.5. Onset samples for *D*1 *onsetsam*_*D*1(1) = *indxD*1(1)
3.6. For *n* = 2 : (*Length of indxD*1 − 1)
If *indxD*1 (*n*) > (*indxD*1 (*n* − 1) + *indxGap*)

o⁢n⁢s⁢e⁢t⁢s⁢a⁢m⁢_⁢D⁢1⁢(n)=i⁢n⁢d⁢x⁢D⁢1⁢(n)

3.7. *onsetsam_D*1 = (*Values of onsetsam_D*1 ≅ 0)
3.8. *tD*1 = Time points in *DataPhoto* corresponding to *onsetsam_D*1
4.
LatD1S1 = (tD1 − tS1) ∗ 1000 in ms.

5. To compute *LatD*2*S*2:
5.1. Define matrix *NegPhoto* containing 1’s and 0’s where 1’s represents positions of negative peaks. *NegPhoto* = *DataPhoto* ≥ *ThD*2
5.2. Substitute [*S*2/*S*1] and [*D*2/*D*1] in steps 3.2 to 4.

#### 2.2.3. Algorithm for CLET using data recorded from the HMD

The notations for HMD-based data are the same as for the LED screen. Also, the inputs are similarly defined, except *ThD*2 which is the threshold of the smaller peaks in between the gaps, as observed in Figure 3B. In this case, *ThD*1 = 180000 and *ThD*2 = 8000.

*II. Steps:*

The algorithm to compute *LatD*1*S*1 here also remains the same as I. Steps 1 to 4 in section Algorithm for CLET using data recorded from the LED screen.

5. To compute *LatD*2*S*2:
5.1. Find all the ordinates of peaks in *DataPhoto*
5.2. Define matrix *xVals* containing 1’s and 0’s where 1’s represents positions of peaks.
5.3. *xVals* = *Replace positions of*   1′ *s with abisccas of DataPhoto*.
5.4. For *n* = 1 : (*Length of signal* – *indxGap*

If xVals (n) Λ xVals (n+1) Λ xVals (n+2) Λ xVals (n+10) < ThD2

Then, the ordinates of peaks with the rest of the values equal to 0 are *yVals*
5.5. Position of small peaks *PosSmPhoto* = *Replace values of yVals* ≠ 0 *with* 1′ *s*.
5.6. Substitute [*S*2/*S*1], [*D*2/*D*1] and [*PosSmPhoto*/*PosPhoto*] in steps 3.2 to 4.

**FIGURE 4:**
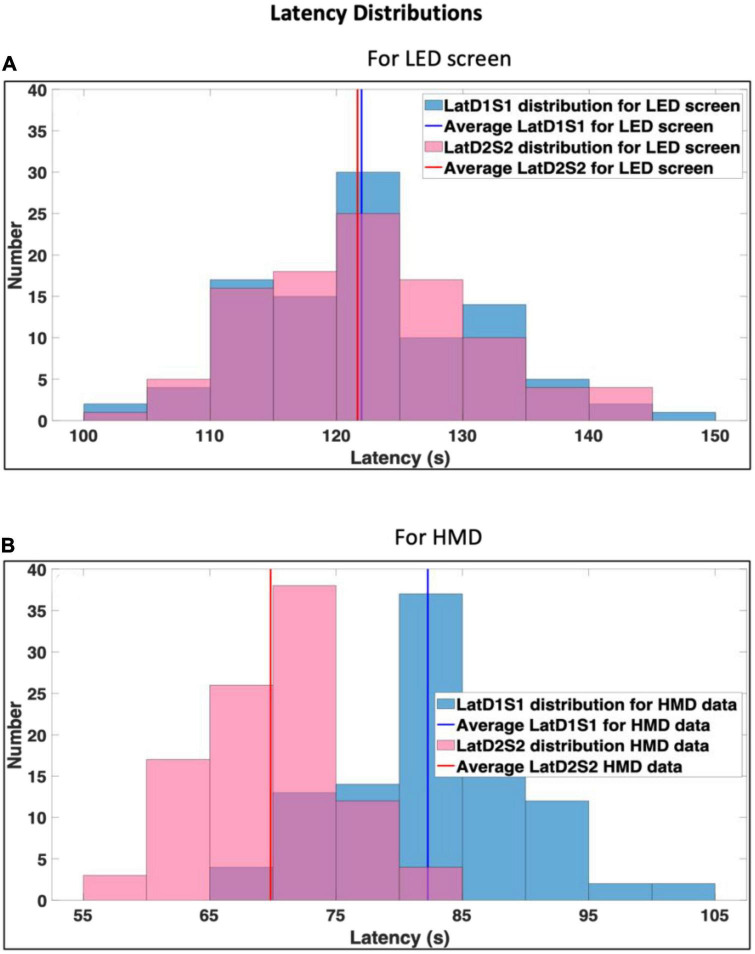
Latency distributions for complete photodiode data recorded from **(A)** Light Emitting Diode (LED) screen and **(B)** head-mounted display (HMD). Blue and pink reflect LatD1S1 and LatD2S2, respectively, while purple reflects overlap in LatD1S1 and Lat D2S2.

The outputs *LatD*1*S*1 and *LatD*2*S*2 computed from steps I and II (Tables available on the GitHub link mentioned in section 10 below) were used to plot distributions for the stimuli set (see Figure 4).

## 3. Results

The Computation of Latencies using the Event-related potential Triggers (CLET) method was successfully implemented and evaluated in two distinct virtual reality (VR) apparatuses, i.e., a non-immersive setup with a LED screen and an immersive setup with a Head-Mounted Display (HMD). The results obtained from both setups demonstrate the efficacy of the proposed CLET approach for accurately aligning EEG/ERP triggers with the presentation of stimuli, thus enabling the extraction and analysis of data with precision.

### 3.1. Latency computation for LED screen

In the non-immersive VR environment with the LED screen (Figure 4A), the CLET method efficiently computed the latencies for a set of white and black stimuli. For the white stimuli, the average latency was 121.98 ± 8.71 ms. Similarly, for the black stimuli, the average latency was 121.66 ± 8.80 ms. The distribution is maximum between 120–125 ms range (Figure 4A). Thus, the consistency in the latencies between the two sets of stimuli indicated the robustness of the CLET approach in this VR configuration.

### 3.2. Latency computation for HMD

In the immersive VR environment with the HMD (Figure 4B), the average latencies were 82.80 ± 7.63 ms, mainly distributed between 80–85 ms, and 69.82 ± 5.52 ms, mainly distributed between 67–77 ms for white and black stimuli sets, respectively. The lower latencies observed with the HMD setup than the LED screen setup suggested faster temporal dynamics for the immersive VR apparatus (Iwama et al., 2022). Although this observation is consistent with findings in the literature (Cattan et al., 2021), variations are particularly subject to protocols that rely on sending triggers for synchronization (Wang et al., 2016; Lees et al., 2018) and specifications of the hardware apparatuses (Rebenitsch and Owen, 2017; Cattan et al., 2018; Andreev et al., 2019; Wilson, 2023).

The results from both VR setups (Figure 4) also highlight the importance of considering latency distributions along with precision and accuracy (Williams et al., 2021) in the computation of latencies for aligning epochs, to avoid any timing discrepancies which would otherwise lead to misinterpretations of data (Cattan et al., 2021; Iwama et al., 2022).

## 4. Discussion

One of the primary strengths of this study lies in the rigorous experimental setup involving the synchronization of various sensors, including EEG, EMG, PPG, photodiode, and nine 3D and a 2D (Miqus) MoCap cameras. This multi-model approach assured a comprehensive investigation of the developed algorithms to compute trigger latencies in VR. Therefore, a direct comparison of this study with the state-of-the-art event-latency computation approaches (Cattan et al., 2018, 2021; Iwama et al., 2022), which were based on lower number of modalities recorded, would be biased. Nevertheless, Figure 4 demonstrates latencies at par with the stated literature. The novelty lies in the two sets of algorithms proposed for the CLET method to accurately detect triggers and compute latencies for both LED screen and HMD data. The adaptability of the algorithms to the subtle variations in the photodiode data shapes for each display type further highlights their versatility and robustness.

The shorter latencies observed in the HMD setup compared to the LED screen setup can likely be attributed to the hardware characteristics of the display technology, which could facilitate faster triggering and data transmission (Cattan et al., 2018; Andreev et al., 2019).

## 5. Conclusion, limitations, and future scope

In conclusion, the results from this research successfully demonstrate the effectiveness of the CLET method for accurately computing latencies in event-related potential (ERP) triggers within two different virtual reality (VR) apparatuses. Efficient synchronization of different sensors and apparatuses also contributed to the validity and the applicability of the CLET method to real-world scenarios. The rapid serial visual paradigm (RSVP) discussed in this study has also been the suggested paradigm due to its simplicity and high temporal accuracy to achieve low values of latencies in triggers (Wang et al., 2016).

A limitation of the proposed algorithms is their semi-automated nature. However, providing open access to the developed codes for CLET, along with novel datasets, tables, and a tutorial video, provides transparency and reproducibility. This encourages the wider scientific community to adopt and validate this method in their own ERP studies, thereby also fostering improvements in the algorithm. One approach could be to use artificial intelligence (AI) or machine learning (ML)-based clustering method(s) to capture transient changes in the shared datasets, followed by automated thresholding to achieve the remaining computation steps as in CLET. Additionally, factors related to the photodiode’s placement and sensor positioning on each display could have influenced the latency measurements. A separate study could be conducted to discuss the best positioning of the photodiode depending on the type of stimuli and display apparatus. It is also stressed that the HMD used in this study consisted of dual displays for each eyepiece. Thus, averaged latency calculated for each lens separately could be used for better accuracy (Cattan et al., 2021). It would be interesting to see the application of CLET in Brain-Computer Interface (BCI) extended to other neuroimaging modalities, such as functional magnetic resonance imaging (fMRI) (Levitt et al., 2023) and magnetoencephalography (MEG) (Liesefeld, 2018), to enable further multimodal investigations of brain activities during Virtual Reality (VR) or Augmented Reality (AR) experiences. This study was limited to only the onset of visual stimuli and offline analysis. With rapid progress in developing VR/AR and haptic technologies (Lm, 2023), accurate computation of trigger latencies will become more critical in real-time BCI feedback systems (Putze et al., 2020; Wen et al., 2021), as well as in transcranial magnetic stimulation (TMS)-based neurorehabilitation (Hernandez-Pavon et al., 2023) where inaccurate triggers could have serious impact on the course of rehabilitation.

## Data availability statement

The datasets, codes, supplementary tables, and a tutorial video are freely available in this link: github.com/BiomedicalEngineeringTechies/CLET.git.

## Author contributions

PS designed research, conducted experiments, developed the algorithm, analyzed the data, wrote the manuscript, and contributed to procuring funds to buy instruments. KG validated the results and co-wrote the manuscript. EV helped to set up the experiment. BV, JN, and XS designed the research and co-wrote the manuscript. AH, TH, and GS contributed to setting up the experiment. GS, JN, and XS contributed to the procurement of funds. XS supervised the study. All authors contributed to the article and approved the submitted version.

## Abbreviations

CLET, computation of latencies in event-related potential triggers; EEG, electroencephalography; ERP, event-related potential; EMG, electromyography; PPG, photoplethysmography; AUX, auxiliary; VR, virtual reality; LED, light emitting diode; HMD, head mounted display; MoCap, motion capture; RSVP, rapid serial visual paradigm; PC, personal computer; QTM, qualisys track manager; BV Rec, brain vision recorder; BCI, brain-computer interface.

## Footnotes

1
github.com/BiomedicalEngineeringTechies/CLET.git
